# An equitable, community-engaged translational framework for science in human lactation and infant feeding—a report from “Breastmilk Ecology: Genesis of Infant Nutrition (BEGIN)” Working Group 5

**DOI:** 10.1016/j.ajcnut.2023.01.020

**Published:** 2023-05-10

**Authors:** Laurie Nommsen-Rivers, Maureen M. Black, Parul Christian, Sharon Groh-Wargo, M. Jane Heinig, Kiersten Israel-Ballard, Julie Obbagy, Aunchalee E.L. Palmquist, Alison Stuebe, Stephanie Merlino Barr, Gabriela V. Proaño, Lisa Moloney, Alison Steiber, Daniel J. Raiten

**Affiliations:** 1 Department of Nutritional Sciences, University of Cincinnati, Cincinnati, OH, USA; 2 Department of Pediatrics, University of Maryland School of Medicine, Baltimore, MD, USA; 3 RTI International, Research Triangle Park, NC, USA; 4 Department of International Health, Bloomberg School of Public Health, Johns Hopkins University, Baltimore, MD, USA; 5 Department of Pediatrics, Case Western Reserve University, Cleveland, OH, USA; 6 Department of Nutrition, University of California Davis, Davis, CA, USA; 7 Department of Newborn and Child Health/Nutrition, PATH, Seattle, WA, USA; 8 Center for Nutrition Policy and Promotion, Food and Nutrition Service, US Department of Agriculture, Washington, DC, USA; 9 Department of Maternal & Child Health, University of North Carolina at Chapel Hill, Chapel Hill, NC, USA; 10 Division of Maternal-Fetal Medicine, University of North Carolina at Chapel Hill, Chapel Hill, NC, USA; 11 Department of Pediatrics, MetroHealth Medical Center, Cleveland, OH, USA; 12 Academy of Nutrition and Dietetics, Chicago, IL, USA; 13 Pediatric Growth and Nutrition Branch, *Eunice Kennedy Shriver* National Institute of Child Health and Human Development, National Institutes of Health, Bethesda, MD, USA

**Keywords:** human milk, lactation science, infant feeding, translational research

## Abstract

Human milk is the ideal source of nutrition for most infants, but significant gaps remain in our understanding of human milk biology. As part of addressing these gaps, the Breastmilk Ecology: Genesis of Infant Nutrition (BEGIN) Project Working Groups 1–4 interrogated the state of knowledge regarding the infant–human milk–lactating parent triad. However, to optimize the impact of newly generated knowledge across all stages of human milk research, the need remained for a translational research framework specific to the field. Thus, with inspiration from the simplified environmental sciences framework of Kaufman and Curl, Working Group 5 of the BEGIN Project developed a translational framework for science in human lactation and infant feeding, which includes 5 nonlinear, interconnected translational stages, T1: Discovery; T2: Human health implications; T3: Clinical and public health implications; T4: Implementation; and T5: Impact. The framework is accompanied by 6 overarching principles: *1*) Research spans the translational continuum in a nonlinear, nonhierarchical manner; *2*) Projects engage interdisciplinary teams in continuous collaboration and cross talk; *3*) Priorities and study designs incorporate a diverse range of contextual factors; *4*) Research teams include community stakeholders from the outset through purposeful, ethical, and equitable engagement; *5*) Research designs and conceptual models incorporate respectful care for the birthing parent and address implications for the lactating parent; *6*) Research implications for real-world settings account for contextual factors surrounding the feeding of human milk, including exclusivity and mode of feeding. To demonstrate application of the presented translational research framework and its overarching principles, 6 case studies are included, each illustrating research gaps across all stages of the framework. Applying a translational framework approach to addressing gaps in the science of human milk feeding is an important step toward the aligned goals of optimizing infant feeding across diverse contexts as well as optimizing health for all.

## Introduction

The “Breastmilk Ecology: Genesis of Infant Nutrition (BEGIN)” Project was designed to: *1*) examine the ecology of human milk, based on the supposition that human milk represents a complex biological system that interacts with both the internal biology and health of the lactating person, the human milk matrix, and the impact on the breastfed infant and external (social, behavioral, cultural, and physical) environments (see Text Box 1 for Core Concepts and Terms); *2*) explore the functional implications of this ecology for both the biological parent and their infant; and *3*) explore ways in which this emerging knowledge can be studied and expanded via a targeted research agenda and translated to support the community’s efforts to ensure safe, efficacious, equitable, and context-specific infant feeding practices in the United States and globally. The matrix of human milk refers to the nutrient and nonnutrient components of foods and their molecular relationships to each other (USDA).

Text Box 1 **Core concepts and terms**

• In the context of this paper, “ecology” is defined as a complex biological system and its interactions with its environment. In this case, the complex system is human milk composition and its inherent biology, and the environment consists of parental and infant inputs and the influence of their respective internal and external environments.
• With due recognition of the need to be observant of issues of gender identity/neutrality, and to improve precision, to the extent possible, for the purposes of the papers described herein, we will use gender neutral terminology where appropriate (e.g., lactating parent/person, etc.), to reflect the reality that not all who lactate identify as female. The term “lactating parent” respects and recognizes those who may have been born female but do not identify as such as well as other gender-relevant contingencies. In situations where reporting primary data (studies/analyses), we will refer to the population as specified (e.g., “the study evaluated 250 lactating mothers”). Moreover, rather than using terms such as “maternal” or “maternal milk,” we will use the terms such as “birthing parent” throughout the report as appropriate as they accurately reflect the biological nature of the birthing parent–infant dyad.
• “Human milk” refers to milk produced by lactating parents and includes both: *1*) breastmilk produced by a parent for their infant and fed directly to infants via the breast or expressed by the lactating parent and then fed to the infant; and *2*) donor/banked human milk produced by lactating persons that is either donated to human milk banks or fed to infants other than their own child.

Alt-text: Text Box 1

The overarching conceptual framework and description of the Project is presented in the BEGIN executive summary [1], the first of 6 manuscripts of this supplement. The subsequent manuscripts in this supplement present the findings of the individual thematic BEGIN Working Groups (WGs) as a continuum of thought that reflects a larger conceptual view of how we can move this important research and public health agenda forward [[2], [3], [4], [5]]. Specifically, the BEGIN Project was accomplished by forming 5 thematic WGs charged with addressing the following themes: *1*) parental factors affecting human milk production and composition; *2*) the components of human milk and the interactions of those components within this complex biological system; *3*) infant factors affecting the matrix, emphasizing the bidirectional relationships associated with the breastfeeding dyad; *4*) the application of existing and new technologies and methodologies to study human milk as a complex biological system; and *5*) approaches to translation and implementation of new knowledge to support safe and efficacious infant feeding practices. This paper represents the results of the deliberations of WG 5.

### Statement of task for WG 5

WG 5 of the BEGIN Project was tasked with developing a framework for translation and implementation of new knowledge in human milk and lactation toward the support of safe and effective infant feeding practices [1]. In Part I of this report, we provide a brief overview of translational science and relevant published translational research frameworks. In Part II, we propose a framework tailored to the unique contributions and challenges of human milk and lactation in optimizing health of the lactating parent–infant dyad. In this section, we also elaborate on a core set of 6 overarching principles that are integral to an ethical and equitable translational framework for science in human lactation and infant feeding. In Part III, we provide a series of case studies applying the proposed framework in addressing important knowledge gaps in human milk and lactation science and their application toward safe and effective infant feeding. We conclude with a summary in Part IV.

## Part I. Translational Science Frameworks

The National Center for Advancing Translational Sciences (NCATS: https://ncats.nih.gov/) was established in 2012 to improve the speed of translation from basic science discoveries to impacting human lives. NCATS defines translation as “the process of turning observations in the laboratory, clinic, and community into interventions that improve health” [6]. Their framework presents 5 interconnected stages that flow between basic research, preclinical research, clinical research, clinical implementation, and public health. NCATS began with the goal of transforming the translational science process “… so that new treatments and cures for disease can be delivered to patients faster” [7]. Science in human lactation and infant feeding would benefit from reducing barriers that impede progress from discoveries to impact. However, the classic NCATS framework is viewed through a disease-curing lens. A lens of health optimization and disease prevention is more appropriate for human lactation and infant feeding.

In the absence of an existing translational framework specific to human lactation and infant feeding, translational frameworks in other fields, including nutrition and dietetics and environmental sciences, may be informative [8]. For example, the National Institute of Environmental Health Sciences (NIEHS) coordinated the development of a highly detailed and complex approach to promote their field’s orientation toward preventive health and research on environmental exposures [9]. The NIEHS framework is presented and described on their website (https://www.niehs.nih.gov/research/programs/translational/framework-details/index.cfm) [10]. Briefly, it incorporates 5 stages of translational research represented by 5 concentric rings with 4 to 6 nodes on each ring. The inner ring encompasses “T1 research” and represents the fundamental questions, addressed through basic science approaches and epidemiology. The next ring encompasses application and synthesis (T2), followed by rings for implementation and adjustment (T3), practice (T4), and impact (T5).

In the broadest sense, human milk and lactation are exposures for the infant and lactating parent, respectively, with the general orientation toward preventing disease and optimizing health. Thus, the NIEHS framework was an attractive starting point for our BEGIN WG, but we desired a simpler template to communicate and disseminate key concepts across an interdisciplinary landscape of human lactation and infant feeding.

Within the environmental health sciences field, Kaufman and Curl [11] proposed a simplified translational research framework for environmental health with stages that span from discovery (T1), health implications (T2), policy/practice implications (T3), policy/practice implementation (T4), and outcome evaluation (T5) in a unidirectional, stage-by-stage manner. Discovery in stage T1 is generated from both basic science and observational studies. Stages T2 and T3 include “integration and cross-fertilization” in an interdisciplinary manner across diverse fields such as epidemiology, toxicology, human clinical research, and biostatistics. Stage T4 encompasses interventions and practice guidelines at both clinical and public health levels. Stage T5 emphasizes assessment of population-level impact.

We were attracted to the Kaufman and Curl framework for its clarity. We also identified areas to revise in conceptualizing a translational research framework for human lactation and infant feeding. We conceptualized the phases as nonlinear to recognize the importance of continuous feedback within and between stages. We also recognized the need to realign and codify descriptors across stages in adapting to the field of human lactation and infant feeding. We received permission from the authors to adapt their template for our report (*personal communication*).

## Part II. A Translational Research Framework for Human Lactation

### Overview of the framework

**FIGURE 1:**
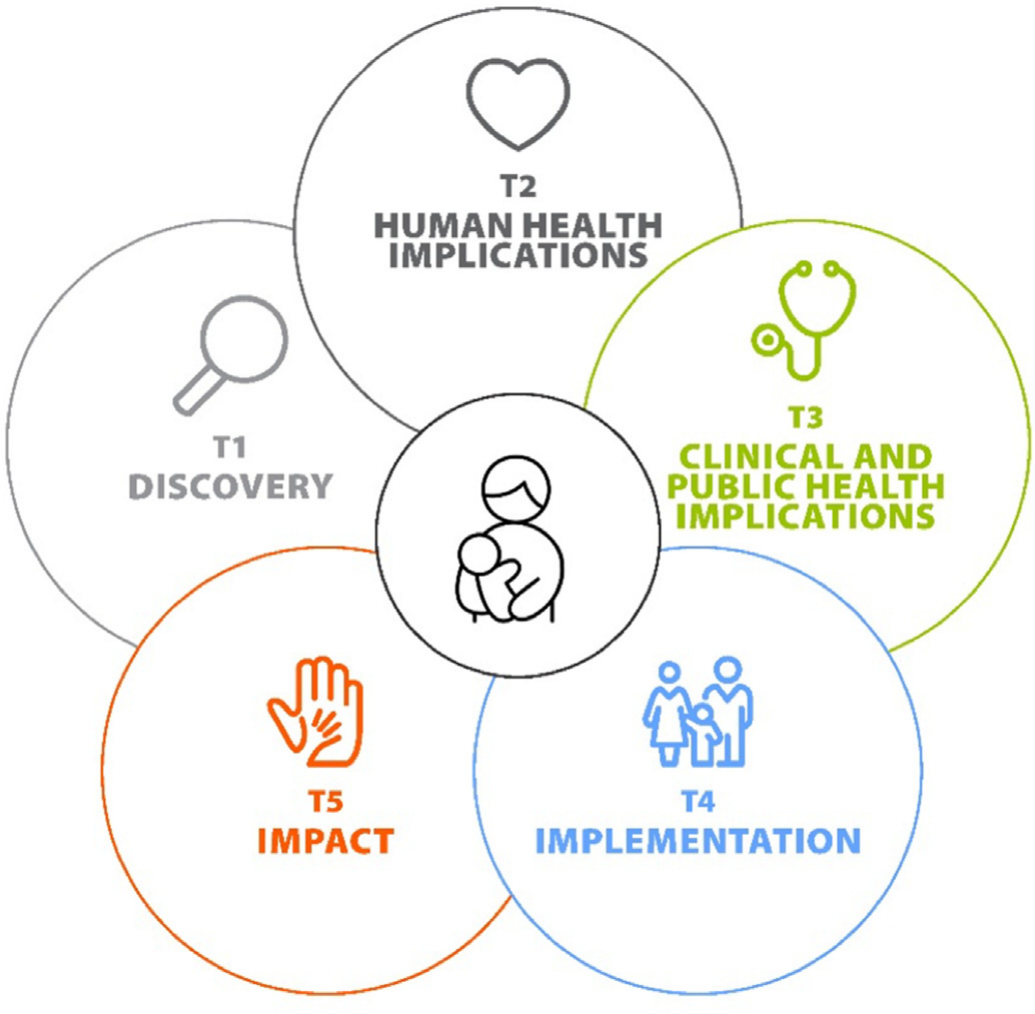
A translational research framework for human lactation and infant feeding.

Figure 1 and Text Box 2 together summarize the components of a 5-stage nonlinear translational framework tailored to human lactation and infant feeding as conceptualized by WG 5.

Text Box 2 **A 5-stage nonlinear translational framework tailored to human lactation and infant feeding as conceptualized by WG 5.**

T1—Discovery

Objective: address the fundamental questions of observing, identifying, and understanding human lactation and infant nutrition at the discovery level.
Approach: cell models and other basic sciences; animal models of lactation and infant nutrition; and observational studies in human cohorts, especially lactating parent–infant cohorts.

T2—Human health implications

Objective: apply discoveries to understand health implications for lactating parents and human milk-fed infants and children.
Approach: highly structured, focused human experiments to establish causation, assess feasibility, develop methods for assessment, or validate prediction tools.

T3—Clinical and public health implications

Objective: scale T2 research findings to test the extent to which new approaches improve outcomes.
Approach: intervention studies to test hypotheses established in T2 research to determine clinical or public health implications in real-world settings. Scaled up intervention studies may include individually or cluster-randomized controlled trials, randomized crossover trials, patient-centered outcomes research, and comparative effectiveness studies.

T4—Implementation

Objective: inform, develop, or implement evidence-based protocols, guidelines, or policies for implementation in clinical, public health, or community settings.
Approach: systematic reviews and meta-analyses, risk communication research, shared decision-making research, and implementation research.

T5—Impact

Objective: utilize epidemiologic surveillance mechanisms to assess attainment of health goals within the health care system, community, nation, region, or globally.
Approach: utilization, expansion, or de novo development of epidemiologic surveillance infrastructure to assess outcomes of relevant interventions and evaluate unintended consequences and demographic disparities in meeting goals.

Alt-text: Text Box 2

The stages of our proposed framework overlay with the mission of the NIH: “…to seek fundamental knowledge about the nature and behavior of living systems (T1, T2) and the application of that knowledge (T2, T3) to enhance health (T3, T4), lengthen life (T5), and reduce the burdens of illness and disability (T5)” [12]. The current knowledge gaps, research needs, and approaches to the study of human lactation and milk biology have been covered by the other BEGIN WGs [[2], [3], [4], [5]] and represent the substance of T1 and T2/T3 of the framework. In Part III, we use case studies to exemplify application of the framework, particularly stages T3 through T5.

### Elaboration of dissemination and implementation research (T4 stage)

The field of breastfeeding and lactation is not immune to the “know-do gap,” the long-recognized lag between knowledge generation and translation into practice [13]. In order to benefit both infants and lactating parents, discoveries in human milk require implementation research to determine how to improve the dissemination and uptake of scientific advancements across diverse populations and contexts [14,15]. Implementation science methods can support the process of adapting research to specific populations and new environments, identify and address implementation determinants, and guide evaluations [16,17]. As the field of implementation science evolves, there has been concerted effort to identify strategies and processes that facilitate successful implementation and sustainment of evidence-based interventions [18,19]. In Text Box 3, we list 9 implementation science clusters, informed by concept mapping of 73 discrete examples, aligned with published strategies in the field of human lactation and breastfeeding [20]. To be effective, the clusters need to “adapt and tailor to the context” [21]. These contextual factors are critical to understand variation in implementation outcomes across populations [22]. Context is elaborated upon later in this section under overarching Principle 3.

Text Box 3 **Examples of applying human lactation and breastfeeding research discoveries to the 9 strategy clusters of implementation science.**

• Use evaluative and iterative strategies
  ➢ Increase the proportion of infants exclusively breastfed at maternity hospital discharge through the development of key driver diagrams and tracking process measures through several iterations of plan-do-study-act cycles [23].
• Provide interactive assistance
  ➢ Leverage pharmacists to address questions regarding medication use during breastfeeding [24].
• Adapt and tailor to context
  ➢ Use formative research to design a context-specific kangaroo mother care intervention [25].
• Develop stakeholder interrelationships
  ➢ Improve breastfeeding initiation and duration in communities with the lowest breastfeeding rates through grassroots peer support [26].
• Train and educate stakeholders
  ➢ Identify and train “breastfeeding champions” at each primary care office within a health care system [27].
• Support clinicians
  ➢ Ensure adequate staffing of lactation support professionals [28].
• Engage consumers
  ➢ Publish commentary submitted by patient advocate groups, such as groups advocating for improving the management of persistently low milk supply [29].
• Utilize financial strategies
  ➢ Incentivize exclusive breastfeeding through modifying the Special Supplemental Nutrition Program for Women, Infants, and Children (WIC) food package for the breastfeeding parent [30].
• Change infrastructure
  ➢ Implement laws to improve workplace breastfeeding support [31].

Alt-text: Text Box 3

### Overarching principles in applying the framework

Our WG identified 6 overarching principles for an equitable, community-engaged translational framework for science in human lactation and infant feeding (Text Box 4).

Text Box 4 **Overarching principles for an equitable, community-engaged translational framework for science in human lactation and infant feeding.**

1. Research spans the translational continuum, moving from one translational stage to another in a nonlinear, nonhierarchical manner.
2. Projects engage interdisciplinary teams in continuous collaboration and cross talk.
3. Priorities and study designs incorporate a diverse range of contextual factors (e.g., biological, evolutionary, geographic, environmental, social ecological, political economic, ethical, legal, and care setting).
4. Teams include community stakeholders from the outset through purposeful, ethical, and equitable engagement that fosters a foundation of trust to prioritize work and optimize impact across translational stages.
5. Research designs and conceptual models incorporate respectful care for the birthing parent and implications for the lactating parent.
6. Research implications for real-world settings account for contextual factors surrounding the feeding of human milk, including exclusivity and mode of feeding.

Alt-text: Text Box 4

#### Overarching Principle 1: Research spans the translational continuum, moving from one translational stage to another in a nonlinear, nonhierarchical manner

The first principle is consistent with the BEGIN premise that human milk is a biological system existing within an ecological mileu of factors that mutually influence one another. As such, this principle recognizes the nonlinear nature of research in human lactation and infant feeding. Progress at any 1 stage informs research priorities at all other stages, whether preceding or following the current stage of research. For example, community-engaged research at the T4 stage may inform priorities at the T1 or T2 stage.

#### Overarching Principle 2: Projects engage interdisciplinary teams in continuous collaboration and cross talk

As illuminated by other BEGIN WGs, human lactation and infant feeding are influenced by, and are influencers of, biology, behavior, and personal environment [[2], [3], [4]]. Interdisciplinary team science includes the full range of contextual factors, including the sociocultural environment, health care providers, and the health care system [20]. Research has the greatest opportunity to impact health through interdisciplinary teams representing expertise across these diverse domains of influence.

A team science approach is a proven strategy for identifying barriers in progressing from discovery to impact and for breaking through narrow disciplinary agendas that dilute the impact of research efforts [32]. Team science is especially key to successfully implementing the middle stages (T2–T4) because high-impact success in these steps requires interdisciplinary teams who are committed to embracing authentic community engagement and mutually value their teammates’ areas of expertise and skills [33]. The end result of team science is high-impact research with rapid progress, facilitated through the translational framework stages [34,35].

#### Overarching Principle 3: Priorities and study designs incorporate a diverse range of contextual factors (e.g., biological, evolutionary, geographic, environmental, social ecological, political-economic, ethical, legal, and care setting)

**FIGURE 2:**
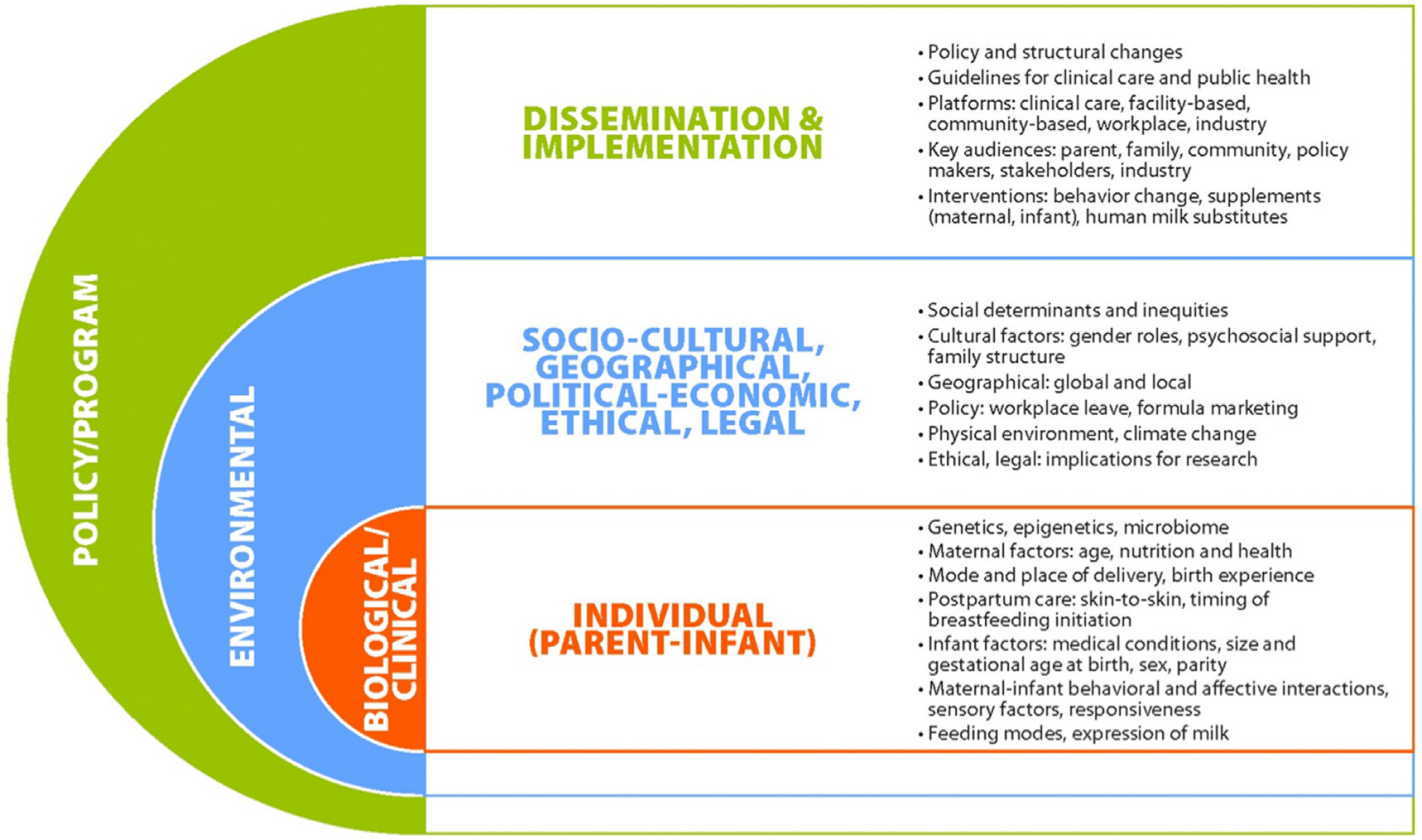
Contextual considerations.

Each stage of our translational research framework incorporates diverse macro- and micro-level contextual factors that influence the translational science journey (Figure 2). The macroscopic contextual factors are the policy and programmatic considerations for implementation, including structural changes, public health initiatives, clinical platforms, and systems dedicated to epidemiologic surveillance, monitoring, and evaluation. The next level encompasses sociocultural, political-economic, geographic, cultural, ethical, and legal factors. Individual level factors include clinical and biological contextual factors. Race is frequently examined as an individual level contextual factor, but race is a social, not a biological construct [36]. As a social construct, race should not be conflated with ethnicity but should be considered within the social determinants of health, as an outcome of structural racism [37]. To illustrate the multiple levels of context, we include 2 scenarios that emphasize contextual factors in Text Box 5.

Text Box 5 **The critical need to consider context.**

Example 1: Understanding infant feeding practices in a low income, rural setting

Biological contextual factors:

• Burden of malnutrition (over-/under-/dual burden)
• Burden of infectious and noncommunicable disease, high prevalence of poor birth outcomes
• Existing public health nutrition interventions such as food fortification, supplement use, etc.

Policy and environmental contextual factors:

• Cultural practices including gender inequity
• Economics including food insecurity
• Physical environment: climate stress including water security and safety
• Access to health systems, public health nutrition programs, and lactation support services
• Dislocation/migration

Example 2: Feeding human milk in a high-income setting

Biological contextual factors:

• Obesity in the birthing parent and associated perinatal morbidities
• High prevalence of delayed secretory activation/excess newborn weight loss
• Medications and recreational substances impacting milk quality and quantity

Policy and environmental contextual factors, with a focus on lactating parent returning to work:

• Chronobiology of human milk composition (changes over a feeding, day, or lactation cycle)
• Direct breastfeeding by the birthing parent compared with feeding of birthing parent’s expressed milk from a bottle
• Economic value of lactation and the provision of human milk
• Local and national policies regarding parental leave, workplace lactation support, childcare
• Equity in access to lactation supportive policies
• Built environment: transportation; proximity of home, childcare, and workplace, etc.

Alt-text: Text Box 5

Infant feeding policies, dissemination and implementation strategies, and epidemiologic surveillance infrastructures [38] are strengthened based on an integrated ecological approach that includes considerations of both the biological and environmental contexts. Strengthening our understanding of human milk as a biological system [39] will enable more granular tailoring of interventions according to the most salient contextual factors across numerous settings. These scenarios entail decision pathways for the parent–infant feeding journey and call for implementation strategies that include psychosocial, nutritional, and structural interventions for both members of the dyad [40]. Context also allows for individualized approaches to infant feeding that respect parental constraints and decision-making autonomy (i.e., those who cannot or choose not to breastfeed).

The lactating parent’s context also informs the full spectrum of translational research; factors that lead individuals to participate in a study can distort associations between these factors in the study sample. This is called collider bias [41]. Within studies of human milk, researchers should take care to thoughtfully develop their conceptual model to account for known sources of bias and take care to ensure that inclusion criteria and incentives for participation do not create spurious associations.

#### Overarching Principle 4: Teams include community stakeholders from the outset through purposeful, ethical, and equitable engagement that fosters a foundation of trust to prioritize work and optimize impact across translational stages

Our framework transcends individual level biological and behavioral interventions to include other domains of influence on health outcomes, ranging from hospital policies to structural factors that perpetuate racial health disparities. Our framework is inspired by the National Institute on Minority Health and Health Disparities (NIMHD) Research Framework [42], which notes that the scientific community has a responsibility to examine hierarchies of power and privilege [43] and engage in practices to mitigate the potential that knowledge production and translation may exacerbate existing social inequities related to racism, political-economic exploitation, and histories of scientific violence [[44], [45], [46]]. Scientific discoveries and innovations accelerate impact when scientists, research teams, research participants, and public stakeholders have diverse lived experiences that are representative of the target population [[47], [48], [49], [50]].

Historical, political-economic, social structural, and institutional barriers can both impede equitable access to scientific training needed to engage in this research and discourage equitable participation of diverse populations in human milk research [51]. Structural barriers within and between societies perpetuate stark inequities between populations that benefit from new scientific discoveries and populations that do not. Thus, historical contexts of human milk research and its translation are also relevant. There is a long history of unethical and exploitative practices in relation to the conduct and translation of research related to infant feeding, human milk, and lactation, which have perpetuated and exacerbated health disparities [[52], [53], [54], [55], [56]]. This history underscores the moral imperative to enact policies, practices, and accountability measures to prevent the exploitation, abuse, and continued marginalization of populations who are typically not supported to engage in the research process. It is critical to include marginalized populations as valued stakeholders and future beneficiaries of knowledge generated through human lactation and infant feeding science.

The core values of diversity, equity, and purposeful stakeholder engagement are foundational to our translational research framework, as these values are critically important to the prioritization of basic research questions, the conceptualization of research study designs, conduct of scientific studies, dissemination to the public, and applications for policy, technology, and industry. Our fourth principle draws upon recommendations that were originally developed through stakeholder engagement with the National Human Genome Research Institute (NHGRI) to elucidate the broader impacts of genomic sciences on society and health [57]. These recommendations have been reproduced in an adapted format in Text Box 6, as they strongly align with ethical and equitable science related to human milk feeding and its translation.

Text Box 6 **Recommendations for the responsible collection and use of samples.**

1. Define community in appropriate and meaningful ways
2. Understand potential benefits and risks for communities and community members
3. Obtain broad community input for all phases of research
4. Respect communities as full partners in research
5. Resolve all issues pertaining to tissue samples
6. Establish appropriate review mechanisms and procedures
7. Facilitate return of benefits to communities, research participants, and populations
8. Foster education and training in community-based research
9. Ensure dissemination of accurate information to the media and public
10. Provide sufficient funding and encourage partnerships

Alt-text: Text Box 6

Adapted from “Report of the First Community Consultation on the Responsible Collection and Use of Samples for Genetic Research,” September 25–26, 2000 [57]

**FIGURE 3:**
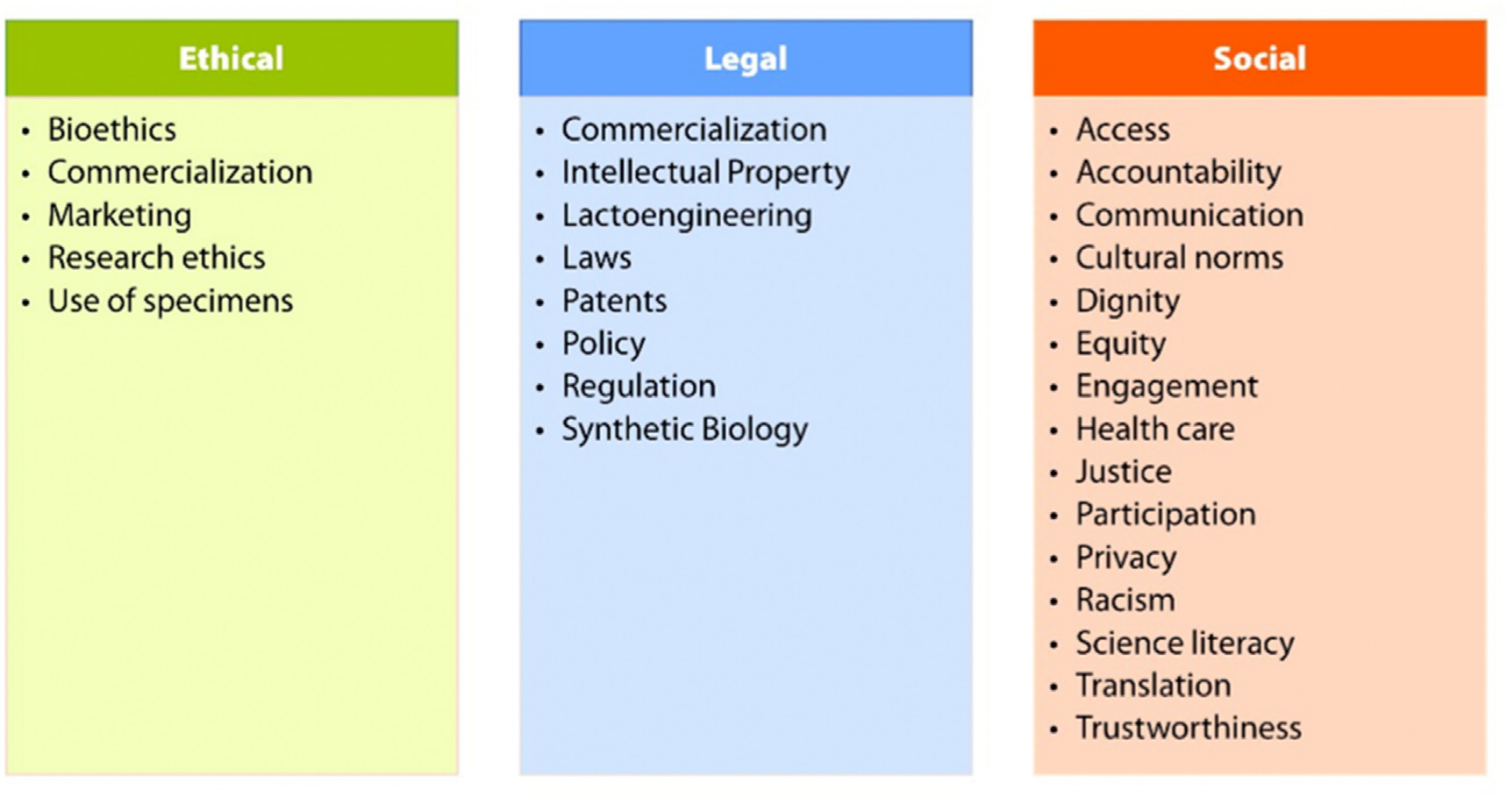
Ethical, Legal, and Social Implications of human lactation and infant feeding research.

Principle 4 emphasizes the ELSI framework of the NHGRI [57]. All scientific research involving human subjects and human biospecimens are grounded by common core values of autonomy, beneficence, nonmaleficence, fidelity and trustworthiness, integrity, veracity, social justice, and respect for dignity [58]. Translating the science of human milk feeding and related discoveries carries with it an additional responsibility to engage diverse stakeholders in the ELSI of the BEGIN project at all stages. Stakeholders may include scientists, research team members, research participants, lactating parents and their communities, health professionals, clinicians, industry, funders, policy makers, and public health practitioners. Examples of the kinds of topics and issues that may be included under each of the ELSI domains are illustrated in Figure 3.

Ethical implications refer to a range of moral dilemmas related to research ethics, bioethics, and the translation of science related to human milk feeding. Ethical implications also encompass issues regarding the alignment of human milk research with core community or societal values. For example, there are emergent ethical issues surrounding the practice of human milk bioprospecting (i.e., research involving the collection of human milk, often among vulnerable global populations primarily for commercialization of human milk compounds) or using scientific discoveries to enhance the global marketing of human milk substitutes. Additional challenges often arise in the informed consent process. For instance, the use of biobanks to store human milk specimens raises unresolved ethical dilemmas regarding how scientists may collect and use milk in current and future research studies. Legal implications may include policies, regulations, accountability structures, and laws that influence the translation and implementation of scientific innovations related to lactoengineering, patents for individual human milk components, and targeted commercialization of human milk-based products for profit. Social implications may include social structural, political-economic, educational, historical, and cultural issues related to the ethical conduct of research, equitable access to participation in research, and the benefits of new discoveries and mitigating potential harm of scientific research and its translation.

Purposeful, ethical, and equitable engagement with diverse stakeholders requires that policies, actions, and accountability strategies are cocreated with research beneficiaries, particularly when beneficiaries are from underserved, underrepresented, or marginalized populations [59]. Implementation of rigorous and transparent ethical standards for the collection of human milk, the use of human milk in scientific studies, and equitable translation and dissemination of research findings are needed. It is also important that translation of this research includes plans to monitor for potential negative impacts on research participants, particularly in marginalized communities and vulnerable global populations. Authentic involvement of beneficiaries (e.g., marginalized racial/ethnic groups; birthing parents in low- and middle-income countries; parents of medically fragile infants) magnifies the public value and impact of the research and bridges disconnections between scientific discoveries and equitable public benefit [[60], [61], [62]].

Research to understand human milk as a biological system holds the potential: *1*) to yield insights into the structure and function of human milk, *2*) to answer questions regarding milk’s role in shaping human biological variation and adaptability across time and space, and *3*) to advance equitable perinatal, postpartum, neonatal, and infant health [56,[62], [63], [64], [65], [66], [67]]. As we embark on a new era in interdisciplinary science related to human milk feeding, we have a responsibility to engage research participants [63], their communities, clinicians, policy makers, and industry in the ELSI of this ambitious endeavor.

#### Overarching Principle 5: Research designs and conceptual models incorporate respectful care for the birthing parent and address implications for the lactating parent

The lactating parent–child dyad comprises a 2-person system. This fundamental truth is a key consideration for translational research in human milk. In 1947, Donald Winnicott observed, “There is no such thing as a baby ... if you set out to describe a baby, you will find you are describing a baby and someone” [68]. Similarly, there is no such thing as human milk without a lactating parent. Lactation affects the health of the lactating parent, and as reviewed by other BEGIN WGs, the health of the lactating parent affects the delivery and composition of human milk to the child [2,3].

The health impact of lactation on morbidity and mortality of the birthing parent is sometimes overlooked. In a Monte Carlo simulation model of breastfeeding rates in the United States, suboptimal lactation rates were associated with 721 excess child deaths (95% CI: 543, 899) and 3340 excess deaths for birthing parents (95% CI: 1886, 4785) [69]. Excess direct medical costs totaled $112,391,907 for children ($110,183,988 to $115,008,632) and $2,601,557,411 for birthing parents ($2,315,439,844 to $2,915,300,320) [69]. These results underscore the importance of understanding the biological effects of lactation on the birthing parent, both during lactation and for long-term parental health.

As emphasized in Principle 4, engagement with lactating parents across the entire translational research continuum is fundamental. Their collaboration contributes to framing research questions and informing the interpretation and application of the results. Discoveries in human milk and lactation translate into improved human health only if they are integrated into the time demands of breastfeeding families. As Tully and Ball write, “Tipping the balancing scales toward biologically optimal feeding necessitates practical, cultural, and emotional support, not stigmatizing discourse” [70]. Parent cocreated translational research studies are needed to successfully integrate research evidence in lactation and infant feeding with parent values and overall family well-being [71].

Although there are structured processes for incorporating research into clinical guidelines, many lactating parents learn about research through lay press reports and other media, including Tweetable and graphical abstracts [72]. However, analyses have shown variability in the research reported by the media, with a heavy influence on press releases. Newspapers are often more likely to cover lower quality observational studies rather than higher quality randomized trials [73]. Media-reported messaging is more likely to reach families with appropriate context if communication is cocreated with stakeholders and lactating parents [74].

Respectful care for the birthing and lactating parent within the health care system is a primary component of Principle 5. “Respectful maternity care” is a universal human right and addresses 7 categories of vulnerability of birthing and lactating parents: physical abuse, nonconsented care, nonconfidential care, nondignified care, discrimination based on patient’s attributes, abandonment of care, and detention in facilities [75]. Providing lactating parents with respectful and dignified care during and after birth is critical in establishing a safe and trusted environment, enabling the parent to feel empowered and to positively affect establishing lactation [76]. This also includes being respectful of parental constraints and autonomy with decision-making related to human milk feeding practices.

#### Overarching Principle 6: Research implications for real-world settings account for contextual factors surrounding the feeding of human milk, including exclusivity and mode of feeding

Science related to human milk feeding benefits greatly from having clear definitions on human lactation and human milk feeding practices and how those definitions might differ from traditional labels related to the lactating birth parent and other lactating individuals. For example, breastfeeding is generally interpreted as feeding human milk to infants directly from the breast [directly from the birthing parent or from another lactating individual (i.e., “wet nursing”)]. However, human milk may be fed to infants by other modes and in combination with a variety of supplementation practices, including expressed and stored human milk (birthing parent’s own milk or donor human milk) fed from a bottle. Discoveries generated from research conducted among infants fed directly and exclusively from the breast do not necessarily translate to populations of infants fed human milk via other modes or nonexclusively, or from a lactating person other than the birth parent. The converse is also true—research conducted with dyads who are not exclusively feeding at the breast may produce outcomes that do not reflect exclusive at-breast feeding. Without clarifying exclusivity and mode of feeding of human milk, the biases are unknown. The potential nonequivalency of not feeding directly at the breast include effects of not experiencing temporal changes in human milk composition throughout a single feeding and throughout the day, loss of bioactivity of human milk components, and loss of the nonnutritive effects of feeding directly at the breast through skin-to-skin contact.

With greater access to consumer grade breast pumps, feeding of expressed human milk is increasingly common. Based on the CDC 2005–2007 Infant Feeding Practices Study II [77], 85% of respondents reported feeding infants expressed breast milk from a bottle. Human milk may be fed freshly expressed and given to the infant or refrigerated or frozen for future feeding. The immunological and nutritional components of human milk are influenced by the storage container, storage conditions, and approach to cooling and warming [78]. Feedings may or may not align with time of day that the milk was expressed, with possible chronobiological effects [79,80]. Expressed human milk may be from the birthing parent, a milk bank, or donor [78,81]. Freeze-drying human milk preserves the stability and may be used in milk banks [82]; quality control for informally shared milk varies tremendously [[83], [84], [85]].

The impact of feeding mode on infant behavior, volume of intake, and duration of providing human milk is an active area of research with inconsistent findings across studies [86]. Feeding infants expressed milk excludes the skin-to-skin contact associated with feeding directly from the breast. The benefits of skin-to-skin contact, including infant attachment, weight gain, development, and well-being, have been attributed to the oxytocinergic system [87]. Feeding expressed breast milk from a bottle without skin-to-skin contact may deny parents and infants these benefits.

Most breastfeeding surveillance does not differentiate by feeding mode [88], including CDC’s National Immunization Survey [89]. For much of the existing research on the effects of human milk feeding, the exposure is operationalized as the provision of human milk, not how or when it was expressed and then fed to the infant, or how it was stored, or processed prior to feeding, or who it came from [90]. The lack of information on the mode of feeding human milk introduces a need to develop and validate updated data collection and epidemiologic surveillance instruments regarding the feeding of human milk, implement revised national and global surveys to gather information on current practices, and develop insights into the trade-offs of feeding modes other than directly at the breast, with the goal of developing context-specific best practice guidelines for feeding human milk [88].

## Part III. Case Studies Applying the Proposed Framework

### Case study A: Applying the framework to dietary guidelines for lactating parents

Since 1980, the Dietary Guidelines for Americans (DGA) have provided science-based advice on what to eat and drink to promote health, reduce risk of chronic disease, and meet nutrient needs [91,92]. Historically, this advice was for individuals 2 y and older and had limited guidance for individuals during pregnancy or lactation. In 2014, Congress passed the Agricultural Act, mandating the DGA include comprehensive guidance for these populations [93]. Consequently, the 2020–2025 DGA provides recommendations for healthy dietary patterns across all life stages—including during lactation [91]. The 2020 DGA edition was informed in part by evidence reviewed by the 2020 Dietary Guidelines Advisory Committee, which describes the state of the science on diet and health, including topics related to nutrition during lactation [94]. The resulting 2020 DGA for lactating parents includes recommended healthy dietary patterns for this life stage, current intakes compared to recommendations, and special dietary considerations.

The Committee recommended research to inform future editions of the DGA, which can be found in the Committee’s Scientific Report (Part D. Chapter 3. Food, Beverage, and Nutrient Consumption During Lactation, and Part E. Future Directions) [94], and within each of the Committee’s Nutrition Evidence Systematic Reviews [95]. In applying the Committee recommendations to our translational framework, nearly all of the identified research needs fall within the T1–T3 stages. We provide examples in Table 1 to illustrate application of the full framework.

**TABLE 1:** Select examples in applying a translational research framework to dietary guidance for lactating parents

Stage	Examples
T1 *Discovery*	Of the 6 systematic reviews focused on nutrition during lactation, the Dietary Guidelines Advisory Committee concluded that insufficient evidence is available to draw conclusions and assign a grade for most research questions. Specifically, strength of evidence grades were not assignable for the relationship between dietary patterns during lactation and the following outcomes: postpartum weight loss, human milk composition (with the exception of lipids, fatty acids, and B12), milk quantity produced, developmental outcomes in the child, and childhood allergic diseases. There was also insufficient evidence to assign grades for most questions examined regarding intake of omega-3 fatty acid supplementation during lactation and child outcomes [94]. Thus, many questions remain to be addressed, including basic and epidemiologic research at the discovery stage [3].
T2 *Human health implications*	Focused human studies are needed to expand our knowledge of how overall dietary patterns and intake of specific foods and nutrients during lactation influence the birthing parent’s health, human milk composition and quantity, and child outcomes.
T3 *Clinical and Public Health Implications*	Building upon T2 research, there is a need for scaled-up randomized trails or quasi-experimental studies to determine the extent to which interventions to improve dietary patterns or intake of specific foods or nutrients will improve human milk quality, human milk quantity, or child health outcomes.
T4 *Implementation*	Research with high engagement of diverse stakeholders is needed to optimally develop programs and policies to ensure equitable access and culturally appropriate adaptation of optimal dietary patterns during lactation across diverse contexts. Figure 2 indicates layers of contexts.
T5 *Impact*	Ongoing epidemiologic surveillance is needed to assess the impact of implemented policies and programs on lactating parents’ diet and health and the health and development of their human milk-fed infants and children. Ongoing epidemiologic surveillance can also reveal disparities in, and possible structural barriers to, optimal dietary patterns during lactation.

### Case study B: Insufficient milk production in the context of obesity

Insufficient milk is one of the most common concerns expressed by lactating parents [96]. Historically, these concerns have been attributed to misperception or mismanagement [97]. However, there is growing recognition of significant knowledge gaps in the causes, prevalence, prevention, and management of physiologic insufficient milk production [2], especially in the context of the current obesity epidemic [[98], [99], [100]]. Table 2 below applies a translational research framework to address some of the knowledge gaps in obesity and milk production in the birthing parent.

**TABLE 2:** Select examples in applying a translational research framework to address obesity-related insufficient milk production

Stage	Examples
T1 *Discovery*	Going back at least 40 y, and as described by the Breastmilk Ecology and the Genesis of Infant Nutrition (BEGIN) Working Group 1, rodent models and epidemiologic research have found associations between birthing parent adiposity and suppressed milk production at the discovery stage [101,102].
T2 *Human health implications*	As reviewed by BEGIN WG 3, 24-h test-weighing and deuterium dilution are well-established validated methods for measuring milk intake in breastfeeding infants [4,103]. However, these methods are not practical in a clinical setting, pointing to the need to develop clinically accessible methods for evaluating birthing parent milk production sufficiency as part of the breastfeeding management toolkit [104,105]. To treat insufficient milk supply, focused studies are needed to elucidate the causal mechanism and pilot potential interventions.
T3 *Clinical and Public Health Implications*	While there is abundant evidence to characterize average milk intake of exclusively human milk–fed infants, clinical management of insufficient milk production requires addressing gaps in knowledge regarding optimal milk volume tailored to individual infant characteristics [106]. Also at this stage, large-scale intervention studies informed by focused pilot studies may lead to effective treatments for insufficient milk production in lactating parents with obesity. Importantly, insufficient milk production can take an emotional toll on new parents, especially given the limited treatment options. Thus, there is a particular need for patient-centered outcomes research that serves those who are at high risk for, or currently diagnosed with, insufficient milk production [71,107].
T4 *Implementation*	Careful messaging is required in addressing physiologic low milk production without exacerbating perceived insufficient milk. Thus, community-engaged research is needed to develop context-specific strategies for dissemination of guidelines for assessing risk and communicating about insufficient milk production in vulnerable patients and implementation of shared decision-making strategies to mutually optimize infant feeding and well-being of the family in affected patients irrespective of how much milk the lactating parent produces [108].
T5 *Impact*	On a population level, there is a gap in research characterizing how broader domains of influence, such as public policy, the built environment, structural inequities, and the health care system impact the prevalence of insufficient milk, such as contributing to the obesity epidemic [109] and worsening metabolic health [[110], [111], [112]], weight stigma [113], xenobiotic exposure [114], and institutional barriers to optimal human milk feeding management and support [115]. Also, ongoing epidemiologic surveillance is needed to monitor the prevalence of insufficient milk and societal level disparities. These research needs will require authentic engagement with key stakeholders, especially parents, in addition to consideration of the other overarching principles described in Part II of this report.

WG, working group.

### Case study C: Cannabis use during lactation

Cannabis is the second most commonly used recreational drug in the United States [116]. Prenatal use is associated with adverse neonatal outcomes in animal models [[117], [118], [119]] and in humans [120,121]. However, prior studies may no longer reflect current risks. Cannabis products used today are 6 to 7 times more potent than in the 1970s [122] and vary widely in type and methods of use. Many prenatal users of cannabis products continue postnatally. The active ingredient, Δ9-tetrahydrocannabinol (THC), transfers into human milk and persists there [120]. Despite significant research efforts spurred by expanding legalization of cannabis products, further work is needed to translate current science into context-appropriate policy, best practices, and clear assessments of risk. In the United States, there is disproportionate criminalization of cannabis use in females of color [123]. Fear of repercussions may limit research participation in these groups, underscoring the need to engage diverse stakeholders across all stages of research involving cannabis and human lactation (Table 3).

**TABLE 3:** Select examples in applying a translational research framework to address cannabis use during lactation

Stage	Examples
T1 *Discovery*	Existing research at the discovery stage includes rodent models, where it has been determined that perinatal cannabinoid exposure may result in lasting deficits in behavior and function [[117], [118], [119]]; for example, cannabinoid exposure in suckling rat pups adversely affects brain maturation and alters early behavior. However, it is not known if cannabis use alters the hormonal milieu of lactation or milk production outcomes in animal models. There is also a gap in large epidemiologic studies at the T1 stage quantifying effects of exposure during lactation on infant outcomes independent of prenatal exposure. Epidemiologic research is also needed to examine outcomes by mode of use, potency, and timing of consumption during lactation.
T2 *Human health implications*	It is known that THC crosses the placenta, and prenatal use affects the fetal brain and has been linked to adverse pregnancy outcomes and adverse outcomes in children [120,124]. Postnatal infant exposure may occur via consumption of human milk or inhalation of secondhand smoke [125,126]. THC is lipophilic and remains detectable in human milk for extended periods of time [127,128], with its concentration influenced by the timing of exposure, human milk fat content, and the parent’s metabolism of the drug [129,130]. However, more in-depth research is needed on the pharmacokinetics of THC and metabolites by mode and potency of parents’ exposure, both in relation to transfer into human milk and uptake and metabolism by the infant. To accomplish these studies, research is needed to develop and validate methods to assess dose and exposure to lactating parents and their infants in diverse contexts.
T3 *Clinical and Public Health Implications*	There are significant gaps in knowledge of the impact of parent cannabis use on parent–infant interactions and related outcomes. Stakeholder-engaged guidelines are needed for the conduct of ethical research in this regard with lactating parents who are already using or exposed to cannabis in their environment. There are also gaps in knowledge regarding cannabis levels in donor human milk, whether procured through human milk banks or informally.
T4 *Implementation*	Most public health entities emphasize counseling and guidance to reduce or end cannabis use rather than cessation of human milk feeding among users [120,130,131]. However, little is known about the impact of these recommendations on use during lactation. For social desirability and sometimes legal reasons, parents may underreport use, making it more difficult to obtain appropriate counseling and care [132,133]. More research is needed in diverse contexts to better understand influences on use of cannabis products and perceptions of safety during lactation. Also, stakeholder-engaged research is needed to develop and test culturally acceptable and effective strategies to support risk communication and shared decision-making regarding cannabis use during lactation and infant exposure in diverse populations. Research is also needed on effective methods to support reduction or cessation of use among diverse populations. Notably, punitive approaches to perinatal use disproportionately harm Black and immigrant parents [133].
T5 *Impact*	Research at the community and societal level is becoming increasingly relevant given the growing number of states that have legalized cannabis use. To understand the population-level impact of these changes in legalization, there is a need to monitor changes in perinatal use patterns and how use in diverse populations is influenced by the dissemination and implementation of strategies and guidelines developed in the T4 stage. Research should also include monitoring of racial and socio-economic disparities in the implementation of ethical, evidence-based care for the lactating parent–infant dyad exposed to cannabis.

THC, tetrahydrocannabinol.

### Case study D: Duration and exclusivity of human milk feeding and the introduction of complementary foods

The first 1000 days of life are critically important for growth and development, and early nutrition exposures have health consequences through all life stages—infancy through adulthood (www.thousanddays.org) [134]. A key consideration pertaining to nutrition during the first year of life is the optimal duration of exclusive human milk feeding and the timing and type of complementary foods to support optimal health of the infant and to lay the foundation for a positive health trajectory. Breastfeeding affords powerful protection against morbidity and mortality, and this protection is maximized during exclusive breastfeeding [135]. However, as new evidence emerges regarding our understanding of human milk composition and lactation biology [2,3], there is a need to periodically re-examine evidence gaps and context-specific research needs regarding exclusive human milk feeding recommendations. In many low- or middle-income country (LMIC) settings, growth faltering during infancy is all too common, and large gaps remain regarding the role of exclusive breastfeeding and complementary feeding in improving growth outcomes in this context [39]. Translational research is needed to test the extent to which strategies designed to optimize outcomes in undernourished populations—such as dietary supplements for the lactating parent or timing of introduction of complementary feeding—improve intergenerational health and well-being.

With regard to infants born in high-income settings, a series of systematic reviews were recently conducted to examine relationships between human milk feeding, complementary feeding, and health outcomes as part of the Pregnancy and Birth to 24 Months Project and the 2020 Dietary Guidelines Advisory Committee work [[136], [137], [138], [139], [140], [141], [142], [143], [144], [145], [146], [147]]. Many evidence gaps were identified in the committees’ reports. Table 4 below exemplifies a translational research framework for addressing some of the knowledge gaps in optimal duration of exclusive human milk feeding (birthing parent’s own milk via breastfeeding, or birthing parent’s own milk or donor human milk via bottle feeding) and the introduction of complementary foods across diverse settings.

**TABLE 4:** Select examples in applying a translational research framework to duration and exclusivity of human milk feeding and the introduction of complementary foods

Stage	Examples
T1 *Discovery*	At the discovery stage, fundamental questions in our understanding of the chronobiology of human milk composition by timing of lactation can inform guidelines on the duration of exclusive human milk feeding (6 mo), types and timing of complementary foods, and feeding practices [39]. As another example, epidemiologic studies across a wide variety of settings are needed to address knowledge gaps in how context (Figure 2) may influence relations between the birthing parent’s nutritional status, duration of exclusive human milk feeding, and health outcomes.
T2 *Human health implications*	Focused studies are needed to identify context-specific interventions regarding the duration of exclusive human milk feeding, composition of complementary foods, and feeding practices that may hold promise in large-scale clinical trials. In alignment with Case Study A, interventions could be designed to examine the interaction between the birthing parent’s nutritional intervention and optimal duration of exclusive human milk feeding.
T3 *Clinical and Public Health Implications*	Clinical trials in diverse contexts (e.g., settings with high rates of undernutrition, or low birth weight; or individual infant or birthing parent characteristics) are needed to determine infant growth and health implications from shorter versus longer duration of exclusive human milk feeding, and from nutritional support for pregnant and lactating parents and various complementary feeding regimens. Long-term follow-up could reveal other implications related to duration of exclusive human milk feeding, such as long-term outcomes for the recipient infant (e.g., immune development, allergic disease, obesity, etc.) and the lactating parent (cardiometabolic health, inter-pregnancy nutritional status and birth interval, economic impacts, etc.).
T4 *Implementation*	Dissemination and implementation research aimed at increasing uptake of current and updated guidelines may include behavior change interventions, communications strategies, and strategies for delivery of nutritional and other interventions during pregnancy and lactation at scale through antenatal and postnatal care platforms. Stakeholder engagement is key to successful design of these studies. Mixed methods research could reveal obstacles (structural, political, environmental, individual, etc.) to sustaining exclusive human milk feeding and/or providing appropriate complementary foods and feeding practices in real-world settings.
T5 *Impact*	Informing guidelines for the optimal duration of exclusive human milk feeding, nutritional support for the lactating parent, and introduction of complementary foods and feeding practices has implications for the health, growth, and development of millions of newborns and nutritional well-being of people who breastfeed, globally. Reduction in infant morbidity and mortality and improvement in linear growth and other outcomes postnatally would be expected. Ongoing epidemiologic surveillance is key to monitoring behavior change and intervention uptake, identifying disparities in these, and measuring impact on health and nutritional outcomes.

### Case study E: Optimizing human milk fortification for very low birthweight infants in high-income country contexts

Human milk is the preferred feeding for nearly all newborns but requires fortification to meet the nutritional needs of preterm, very low birthweight infants (VLBW, infants born less than 1500 g) [148]. Human milk offers many advantages, including a unique nutritional composition, improved feeding tolerance [149], and decreased incidence of several life-threatening complications of prematurity [[150], [151], [152], [153]]. Despite its numerous benefits, there are many challenges in providing human milk to the VLBW infant. Lactating parents may struggle to sustain adequate human milk volume during the weeks, and often months, of hospital-induced separation. This results in the frequent need for supplementation with donor human milk, which carries its own challenges including potential for poor growth [154], supply chain issues, and the disruption of nutritive and nonnutritive human milk components in donor human milk processing [155,156]. Additionally, human milk cannot meet the significant protein and mineral needs of VLBW infants, and inadequate fortification may increase the risk of poor outcomes [157,158]. Targeted fortification is an innovative, individualized approach that uses human milk analysis to inform macronutrient targets for feeding human milk to VLBW infants, rather than *assuming* the macronutrient concentration of human milk and using a standard fortification plan. Recently, a mid-infrared human milk analyzer was approved by the US Food and Drug Administration for clinical use [159].

**FIGURE 4:**
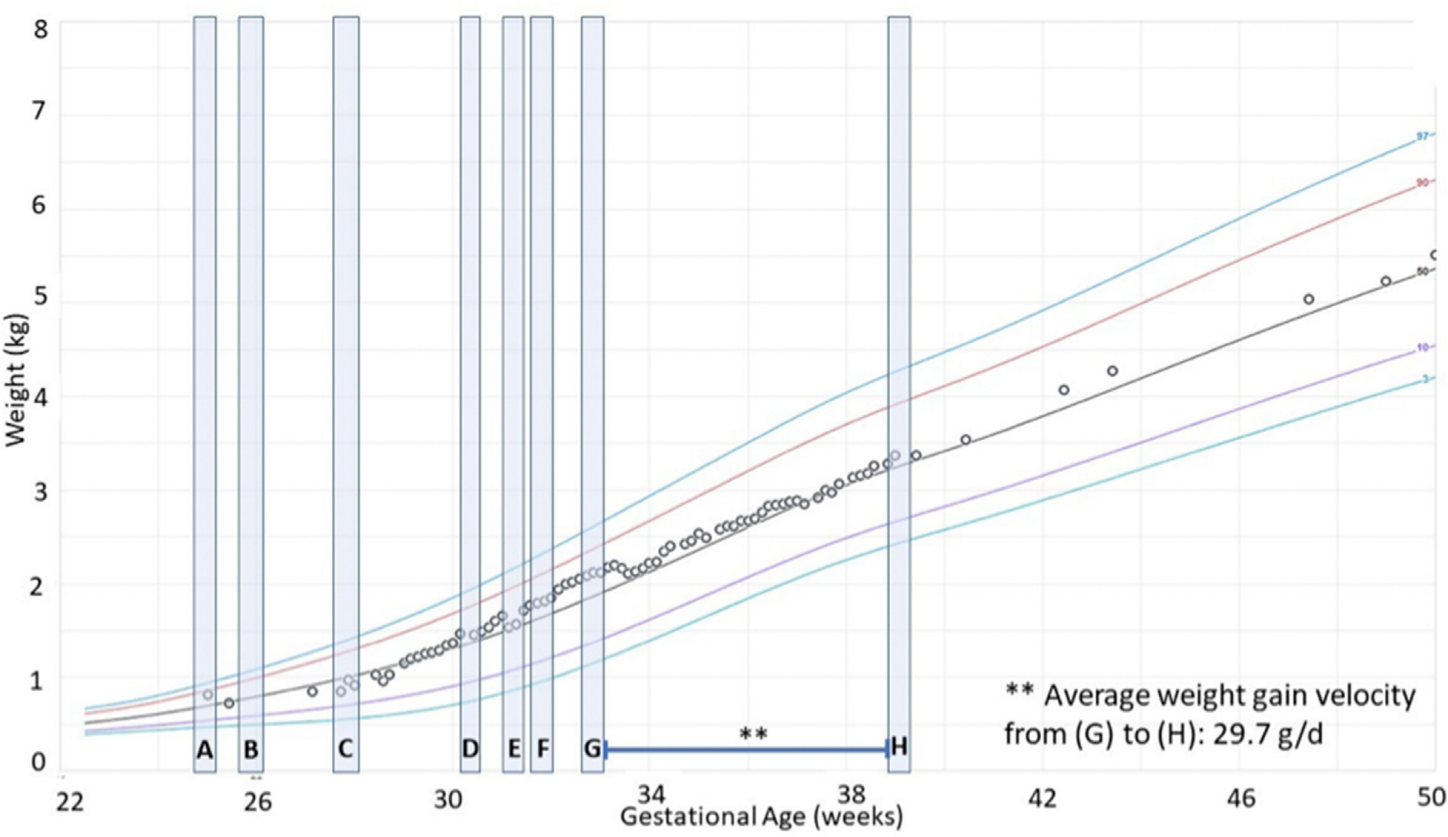
Growth trajectory for Baby L. Case Study Baby L. The Fenton growth chart depicts Baby L’s neonatal intensive care unit journey, highlighting nutrition milestones [162]. (A) Baby L was born at 25 0/7 wk gestational age, weighing 810 g, with parenteral nutrition initiated on the first day of life; (B) Weekly human milk analysis using mid-infrared spectroscopy was initiated; (C) Enteral feedings of expressed milk from the birthing parent were initiated; (D) Half fortification (1:50) using a concentrated liquid human milk fortifier with hydrolyzed protein was initiated; (E) Parenteral nutrition discontinued; (F) Full fortification (1:25) was initiated, with enteral feeds providing 150 mL/kg, 120 kcal/kg, and 3.8 g/kg protein; (G) Targeted fortification initiated using a liquid protein hydrolysate, with enteral feeds providing 150 mL/kg, 125 kcal/kg, and 4.3 g/kg protein; (H) Baby L was discharged from the NICU at 39 0/7 wk postmenstrual age, on a combination of expressed milk from the birthing parent and preterm infant formula due to a diminished supply.

A case of a VLBW infant born at a level III neonatal intensive care unit in the United States is presented to illustrate the use of targeted fortification in a clinical setting and present examples of research needs for further optimizing human milk fortification within the context of the proposed translational framework. Baby L was born at 810 g and 25-wk gestation. In accordance with the NICU’s guideline, human milk analysis was initiated at approximately 2 wk of life, enabling all colostrum to be administered to the infant and confirming milk supply was sufficient to support a targeted fortification protocol. Once human milk analysis was initiated, a weekly representative sample was collected by obtaining an aliquot from a 24-h pooled batch of the birthing parent’s milk. From this weekly analysis a weekly nutrition plan was created to meet the targeted fortification goals for energy and protein intakes, with targeted fortification providing 0.5 g/kg/d more protein compared to standard fortification. Implementation of this nutrition plan is illustrated in Figure 4. Baby L had a successful hospital stay and achieved optimal weight gain (Figure 4) and overall growth (not shown). However, to scale up implementation of targeted fortification, substantial research gaps must be addressed, as exemplified in Table 5 [157,160,161].

**TABLE 5:** Select examples in applying a translational research framework to targeted fortification for VLBW infants

Stage	Examples
T1 *Discovery*	Examples at the T1 stage include: *1*) epidemiologic investigations of variability in milk quality, including macronutrient and micronutrient content, of parental and donor human milk and *2*) basic science research to identify promising protein sources for fortification of human milk in VLBW settings.
T2 *Human health implications*	At the T2 stage, needs include the development of novel approaches to designing ethical clinical trials considering the highly vulnerable population. Examples include: *1*) improved technology to enable the use of micro volumes of human milk in research; *2*) optimal human milk aliquoting strategies for adequate representativeness with minimal volume in targeted fortification protocols [161]; *3*) clinical trial designs that do not entail randomization to known inferior NICU feeding protocols outside of the standard of care; and *4*) novel approaches to improving the availability of the birthing parent’s milk to NICU infants.
T3 *Clinical and Public Health Implications*	At the T3 stage, research examples include: *1*) clinical trials examining the optimal human milk nutrient composition according to infant disease state, birth weight, and gestational age; *2*) comparative effectiveness research of targeted versus standard fortification protocols according to infant birth weight and gestational age; and *3*) patient-centered outcomes research aimed at improving the lactating parent experience in initiating and sustaining milk expression.
T4 *Implementation*	As targeted fortification becomes more widespread, research needs at the T4 stage include: *1*) systematic reviews at regular intervals to inform evidence-based protocols for the nutritional care of VLBW infants [163] and *2*) research regarding role delineation and best practices training among NICU dietitians, nurses, lactation consultants, and physicians in supporting diverse populations of lactating parents, managing the provision of banked donor human milk, and implementing evidence-based nutritional care plans for VLBW infants [164,165].
T5 *Impact*	At the T5 stage, ongoing epidemiologic surveillance is needed to monitor the impact of evolving nutritional care protocols for VLBW infants and identify disparities in implementation of best practices across demographic and racial groups.

NICU, neonatal intensive care unit; VLBW, very low birth weight.

### Case study F: Provision of human milk to small sick newborns in low- and middle-income countries

Globally, 2.5 million newborns die in the first 28 d of life, with around 98% of these deaths occurring in LMICs and nearly 80% concentrated in either sub-Saharan Africa or Southern Asia [166]. At greatest risk of death are small and sick newborns (SSNBs), defined as those born preterm (<37 wk gestation), small for gestational age, low birth weight (<2500 g), or suffering an illness from a birth complication and requiring neonatal hospitalization [167]. The World Health Organization states that while providing the birthing parent’s own milk is the foremost recommended option for feeding SSNBs, when this is not possible, provision of safe donor human milk from a human milk bank is the preferred alternative to infant formula [168]. In the resource-constrained settings of many LMICs, preterm infant formula is often not available, underscoring the essential role of human milk in nourishing SSNBs in these settings [[169], [170], [171], [172], [173], [174], [175], [176], [177], [178]]. However, there are significant challenges in achieving human milk feeding for SSNBs [179]. Challenges in accessing the birthing parent’s own milk include the immature or delayed neuro-cognitive capabilities of SSNBs resulting in difficulty feeding at the breast [180]. Additionally, the majority of SSNB hospital settings in LMICs limit birthing parent–infant contact and interaction, compounding often traumatic circumstances surrounding a preterm birth, resulting in high levels of parental stress [181], delayed secretory activation, and difficulty sustaining lactation. Challenges in accessing donor human milk include lack of human milk banks. Currently an estimated 800,000 infants receive donor human milk from over 750 human milk banks from 66 countries around the world, most of which are in mid- to high-income countries [182]. Expansion of human milk banks in LMIC settings has been hindered by lack of research to inform the adaption of technologies for donor human milk processing, screening, storage, and transport that are appropriate for LMIC resource-constrained health systems. Additionally, establishment of human milk bank operational and ethical standards, as well as comparative effectiveness research regarding feeding outcomes and cost-effectiveness, is urgently needed for LMICs [183,184], where the greatest burden of SSNB exists. Research to specifically target the special needs of SSNBs must build upon the inclusion of the birthing parent’s experience to ensure “respectful maternity care” and equitable access to that parent’s own milk or safe donor human milk for all infants. Table 6 below provides examples across our translational research framework for addressing the numerous gaps in optimizing access and intake of human milk for the SSNB in LMIC settings.

**TABLE 6:** Select examples in applying a translational research framework to provision of human milk to small sick newborns in low- and middle-income countries

Stage	Examples
T1 *Discovery*	At the T1 stage, research is needed to assess variation in composition of donor human milk from the birthing parent in low-, middle-, and high-income settings; to characterize how quality of donor human milk varies according to milk banking screening, pooling, pasteurization, storage, and delivery methods specific to human (not bovine) milk or alternative low-cost treatment systems; and to develop low-cost, point-of-care DHM screening mechanisms.
T2 *Human health implications*	At the T2 stage, there is need for innovative research to support long-term lactation for birthing parents of SSNBs. Examples include: *1*) development of LMIC-appropriate hospital-grade breast pumps; *2*) development of improved technologies for feeding SSNBs expressed human milk in LMIC settings that optimize quality and composition of human milk reaching the gut of SSNB (and reduce fat loss due to adhesion to tubing); and *3*) developing respectful models of care for the birthing parent-SSNB dyad through feasibility studies such as an enhanced training model for hospital staff to provide specialized lactation support, or a family participatory care model, or a peer group support model. Innovative research is needed to adapt human milk bank systems to LMIC settings, including development and evaluation of LMIC-appropriate point-of-care donor screening mechanisms, and DHM processing and storage protocols, and the development and pilot testing of models for integrating LMIC-specific human milk bank systems into newborn care and human milk feeding promotion.
T3 *Clinical and Public Health Implications*	At the T3 stage, research includes clinical trials in LMIC settings of the most promising technology innovations and maternity interventions to emerge from T2 research aimed at improving lactation support, exclusive human milk feeding at discharge, and appropriate use of DHM and assessing reduction in neonatal morbidity and mortality and reduced length of hospital admission.
T4 *Implementation*	At the T4 stage, research needs include development of “best in practice” guidelines for SSNB care in LMIC settings and implementation science research. Examples of DHM research needs include: *1*) determining motivations, barriers, and trade-offs for donating, selling, sharing, and receiving DHM in LMIC settings; *2*) development of evidence-based global standards for ethical, safe, and effective human milk banking operations; and *3*) systematic reviews and analyses on the requirements and cost-effectiveness of an LMIC-appropriate comprehensive HMB model to inform sustainable expansion.
T5 *Impact*	At the T5 stage, ethical epidemiologic surveillance frameworks are needed to monitor lactation support to the birthing parent, the human milk donor, and human milk recipient, with consideration for vulnerability, equity, and fairness; respect for autonomy; and human rights. Examples of needed epidemiologic surveillance include: *1*) global feeding practices of SSNBs by conducting a multi-country assessment; *2*) improving routine indicators to enable monitoring of lactation support, inpatient SSNB feeding practices, and neonatal health outcomes; *3*) document expansion of human milk banks in LMIC settings; utilization of standards; and impact on human milk intake, length of hospital stay, and ultimately, the impact on neonatal health, morbidity, and mortality; and *iv*) ongoing epidemiologic surveillance of geographic, economic, and racial inequities in SSNBs receiving human milk.

DHM, donor human milk; HMB, human milk bank; LMIC, low- or middle-income country; SSNB, small and sick newborn.

## Part IV. Summary and Conclusions

Translational research frameworks were initially established to accelerate “bench to bedside” progress in curing disease. A framework that is reoriented toward optimizing health of the lactating parent–infant dyad has potential for accelerating reach and impact of human milk and lactation research and extending progress from bench to community-based lactating parents and infants. In our report, we present several overarching considerations in the conduct of ethical and equitable research within the lactating parent–human milk–infant ecosystem. As we illustrate in our series of case studies, research gaps exist across the entire spectrum of our translational research framework. Eliminating disparities in health outcomes and optimizing health for all will require authentic commitment to stakeholder engagement, adequate funding for interdisciplinary collaboration, valuing of the critical “middle stages” that strengthen the bridges between discovery and impact of human milk research, and implementation research to inform effective dissemination in collaboration with stakeholders.

## Funding

The BEGIN Project was initiated by the Pediatric Growth and Nutrition Branch of the *Eunice Kennedy Shriver* National Institute of Child Health and Human Development (NICHD) of the National Institutes of Health in partnership with the Bill & Melinda Gates Foundation and the Academy of Nutrition and Dietetics (Academy). The publication of this supplement was made possible by the NICHD, and support for assistance (by BioCentric, Inc.) with editing, proofing, and submitting the manuscripts was also provided by the NICHD.

## Acknowledgements

We thank the *Eunice Kennedy Shriver* National Institute of Child Health and Human Development, specifically Andrew Bremer, MD, PhD, MAS, along with the Academy of Nutrition and Dietetics for their support. Additionally, we thank Kenneth Sherr, PhD, MPH for his critical review and expert feedback regarding implementation research. We would like to dedicate this paper to James E. Heubi, MD, who was the founder and director of the University of Cincinnati NIH-funded Center for Clinical and Translational Science and Training. Dr. Heubi provided great insights regarding the translational research framework before sadly passing away in August of 2021. We acknowledge the role of BioCentric, Inc. (Collingswood, New Jersey) and its staff (particularly Kevin Jarvis, PharmD, and Andrea Tucker, MA, ELS) in editing and formatting the manuscript in accordance with the journal style and assisting with the manuscript submission process.

## Author contribution

The authors’ responsibilities were as follows – DJR: conceptualized the project; all authors contributed to writing the manuscript. Introduction led by DJR; Part I led by LNR; Part II led by LNR, PC, AELP, AS, KIB, MMB, and MJH; Part III led by JO, LNR, MJH, PC, SMB, and SGW; and Part IV led by LNR. LNR: prepared the final draft of the manuscript, and all authors: read and approved the final manuscript.

## Conflicts of interest

All authors have declared no conflicts of interest related to existing financial arrangements with any company or organization sponsoring this manuscript. There was no committed funding for the writing of this manuscript for any author.
